# Helicase Domain Encoded by *Cucumber mosaic virus* RNA1 Determines Systemic Infection of *Cmr1* in Pepper

**DOI:** 10.1371/journal.pone.0043136

**Published:** 2012-08-15

**Authors:** Won-Hee Kang, Jang-Kyun Seo, Bong Nam Chung, Kook-Hyung Kim, Byoung-Cheorl Kang

**Affiliations:** 1 Department of Plant Science, Plant Genomics and Breeding Institute, and Research Institute for Agriculture and Life Sciences, Seoul National University, Seoul, Korea; 2 Department of Agricultural Biotechnology, Seoul National University, Seoul, Korea; 3 National Institute of Horticultural and Herbal Science, Rural Development Administration, Suwon, Korea; Soonchunhyang University, Republic of Korea

## Abstract

The *Cmr1* gene in peppers confers resistance to *Cucumber mosaic virus* isolate-P0 (CMV-P0). *Cmr1* restricts the systemic spread of CMV strain-Fny (CMV-Fny), whereas this gene cannot block the spread of CMV isolate-P1 (CMV-P1) to the upper leaves, resulting in systemic infection. To identify the virulence determinant of CMV-P1, six reassortant viruses and six chimeric viruses derived from CMV-Fny and CMV-P1 cDNA clones were used. Our results demonstrate that the C-terminus of the helicase domain encoded by CMV-P1 RNA1 determines susceptibility to systemic infection, and that the helicase domain contains six different amino acid substitutions between CMV-Fny and CMV-P1_._ To identify the key amino acids of the helicase domain determining systemic infection with CMV-P1, we then constructed amino acid substitution mutants. Of the mutants tested, amino acid residues at positions 865, 896, 957, and 980 in the 1a protein sequence of CMV-P1 affected the systemic infection. Virus localization studies with GFP-tagged CMV clones and *in situ* localization of virus RNA revealed that these four amino acid residues together form the movement determinant for CMV-P1 movement from the epidermal cell layer to mesophyll cell layers. Quantitative real-time PCR revealed that CMV-P1 and a chimeric virus with four amino acid residues of CMV-P1 accumulated more genomic RNA in inoculated leaves than did CMV-Fny, indicating that those four amino acids are also involved in virus replication. These results demonstrate that the C-terminal region of the helicase domain is responsible for systemic infection by controlling virus replication and cell-to-cell movement. Whereas four amino acids are responsible for acquiring virulence in CMV-Fny, six amino acid (positions at 865, 896, 901, 957, 980 and 993) substitutions in CMV-P1 were required for complete loss of virulence in ‘Bukang’.

## Introduction

Plant viruses elicit various resistance responses in plants. One of the best known responses is mediated by resistance (R) genes in plants and avirulence genes in plant viruses [1]. Due to error-prone RNA polymerase activity, a short replication cycle, and a large number of genomes in a single cell of the host, plant viruses evolve rapidly [2]. These viral characteristics induce frequent genetic variation, including mutation and recombination. The high mutation rate observed in plant viruses results in the appearance of resistance-breaking viral strains. Plant viruses can use two approaches to elicit resistance breaking in plants: mutation and/or recombination in the avirulence gene of the pathogen, and the virulent variants then spread within the agro-ecosystem [3]. Therefore, identification of avirulence determinants is important for understanding coevolution between plant R genes and virus avirulence genes, and for developing durable virus-resistant cultivars.

*Cucumber mosaic virus* (CMV) has the broadest host range among plant viruses. CMV is a member of the genus *Cucumovirus* in the family *Bromoviridae*. The icosahedral CMV particles contain single-stranded positive-sense RNA and tripartite genomes that consist of RNA1, RNA2, and RNA3 [4]. RNA1 and RNA2 encode the 1a and 2a proteins, respectively. These two proteins are involved in replication of the viral genome [4]. The 1a protein contains two functional domains: the N-terminal methyltransferase domain and the C-terminal helicase domain [5], [6]. The 1a protein has been implicated not only in replication but also in the regulation of systemic infection [4]. The 2a protein has RNA polymerase activity and is associated with a membrane-bound RNA-dependent RNA polymerase (RdRp) [7]. RNA3 encodes the 3a movement protein (MP), which is essential for movement of viral RNA from cell to cell [8]. RNA4, which is a subgenomic RNA derived from RNA3, encodes the coat protein (CP) [9]. Subgenomic RNA4A from RNA2 encodes the 2b protein, which has a partially overlapping open reading frame (ORF) with the 3′ end of the RNA2 sequence encoding the 2a protein [10]. The 2b protein affects the host range and acts as a suppressor of post-transcriptional gene silencing [11], [12].

The avirulence determinants of CMV have been mapped to different RNAs, depending on the resistance mechanisms. RNA1 was determined to be an avirulence determinant of the hypersensitive response in tobacco [13], [14], local and systemic movement in squash [15], and replication of satellite RNA in squash [16], [17]. Avirulence determinants involving RNA2 were identified by induction of the hypersensitive response in cowpea [18], [19] and hypervirulence and systemic movement in tobacco [11], [20], [21], [22]. RNA3 is involved in the limitation of movement between epidermal cells [23], systemic movement in cucurbits, maize, and tobacco [24], [25], and the hypersensitive response in tobacco [26].

During the last decades, various sources of resistance to CMV have been identified by pepper breeders. However, most sources have only partial resistance controlled by multiple genes [27], [28], [29], [30], [31]. Inheritance studies of these sources demonstrated that the inheritance of each source is controlled quantitatively [27], [28], [29], [30]. Recently, we reported a new resistance source, *Capsicum annuum* ‘Bukang,’ which contains a single dominant resistance gene (*Cmr1*) [32]. ‘Bukang’ is a commercial pepper cultivar resistant to CMV-P0 strains (CMV-Kor and CMV-Fny). However, a new isolate, CMV-P1, capable of breaking the *Cmr1*-mediated resistance, was identified in Korea [33]. This study was conducted to identify the virulence determinant of CMV-P1.

## Materials and Methods

### Plant and Virus Materials

*Capsicum annuum* ‘Bukang’ was used for the observation of disease responses to CMV. ‘Bukang’ is a commercial cultivar (Monsanto Korea Inc., Cheongwon-Gun, Korea) known to contain the CMV resistance gene, *Cmr1* [32]. The full-length infectious cDNA clones of CMV-Fny developed in our previous study [34] were used as a virus source.

### Construction of Chimeric Viruses and Amino Acid Substitution Mutants

The full-length cDNA clones of CMV were cloned into the pSNU1 vector [34]. To construct infectious cDNA clones of CMV-P1, full-length cDNAs of CMV-P1 RNA1, RNA2 and RNA3 were amplified by RT-PCR using appropriate primer pairs (CMV-R1R2-5′-BamHI-Fw and CMV-3′-BamHI-Rv to amplify RNA1 and RNA2; CMV-R3-5′-BamHI-Fw and CMV-3′-BamHI-Rv to amplify RNA3) (Table S1) and ligated into the pSNU1 binary vector [34] digested with *Bam*HI. The resulting cDNA clones were named pP1, pP2 and pP3, respectively. To construct CMV RNA2-based agroconstruct expressing GFP upon viral replication, the full-length CMV RNA2 cDNA region of C2-A1-EGFP ([35]; generously provided by Dr. Jin-Sung Hong, Seoul Women’s University, Korea) was amplified by PCR using a primer pair (CMV-R1R2-5′-BamHI-Fw and CMV-3′-BamHI-Rv) (Table S1) and inserted into the pSNU1 binary vector digested with *Bam*HI. The resulting cDNA clones were named pCYR2Δ2b-GFP. The 2b coding sequence of CMV was C-terminally fused with GFP coding sequence. Digestion, ligation and polymerase chain reaction (PCR) overlap methods were used to construct chimeras and amino acid substitution mutants [36]. The restriction sites used and their nucleotide positions within the RNA1 genome sequence of CMV-Fny are *Eco*RI (1), *Mfe*I (1895), and *Bam*HI (3357). Five chimeras, F1/P1.a, F1/P1.b, F1/P1.c, F1/P1.d.e and F1/P1.d, were constructed by the digestion and ligation method using the *Mfe*I, *Eco*RI and *Bam*HI restriction enzyme sites. P1/F1.d.e constructed by the digestion and ligation method using the *Sal*I restriction enzyme site from the CMV-P1 sequence. To construct amino acid substitutions in CMV-Fny RNA1, PCR-based site-directed mutagenesis was used. PCR was performed between the *Mfe*I and *Bam*HI sites using mutagenic primers that result in amino acid mutations. PCR amplicons were ligated into the pGEMT (Promega, Madison, WI, USA) or TOPO (Invitrogen, Carlsbad, CA, USA) TA cloning vectors. After sequence validation, subcloned inserts and CMV-Fny RNA1 were digested with *Mfe*I and *Bam*HI and ligated. To introduce multiple point mutations, the constructed mutant clones were used as templates to prepare mutagenic primers. All chimeric virus constructs were transformed into *Agrobacterium tumefaciens* strain GV2260.

### Virus Inoculation

The *Agrobacterium* containing each CMV construct was inoculated into *Nicotiana benthamiana,* and CMV-infected *N. benthamiana* plants were used as the inocula for *Capsicum* plants. *A. tumefaciens* containing CMV constructs were grown in YEB media containing kanamycin (50 µg/ml) and rifampicin (25 µg/ml) at 30°C. Each *Agrobacterium* strain was grown to an OD_600_ of 0.5, and CMV RNA1-, CMV RNA2-, and CMV RNA3- containing strains were mixed at a 1∶1:1 ratio. The cell suspension in 100 mM MES/100 mM MgCl_2_ buffer (pH 5.5) was incubated at room temperature for three to five hours. Then, the cell suspension was inoculated into *N. benthamiana* using a 1-ml syringe. At 10–14 days post-inoculation (dpi), buffered extracts from systemically infected leaves of *N. benthamiana* were inoculated mechanically onto the cotyledons of ‘Bukang’ seedlings using Carborundum. Inoculated seedlings were kept in a growth chamber at 23 to 25°C until symptoms appeared. The nature of the progeny of the chimeric viruses was verified by direct sequencing.

### Detection of CMV Accumulation by ELISA

Sixteen to 28 days after inoculation, the CP of CMV was detected by an enzyme-linked immunosorbent assay (ELISA), conducted in accordance with the manufacturer’s protocol (Agdia, Elkhart, IN, USA). Two leaf discs of CMV-infected ‘Bukang’ were used for ELISA analysis. Three independent experiments each were performed in triplicate. Each sample was measured at an absorbance value of 405 nm in an ELISA reader (Anthos, Eugendorf, Austria).

### Observation of Virus Infection Using GFP

The *A. tumefaciens* strain containing one of the various CMV constructs capable of expressing the green fluorescent protein (GFP), CMV-Fny-GFP, CMV-P1-GFP, F (-iii, -vi)-GFP, or F (iii, vi)-GFP, was used to monitor the systemic spread of CMV. Buffered extracts of *N. benthamiana* leaves infected with various constructs were inoculated onto ‘Bukang’ cotyledons as described above. At two to six dpi, GFP was detected using a confocal laser-scanning microscope (Carl Zeiss-LSM510, Jena, Germany). The optimal brightness and contrast of all images were enhanced using Adobe Photoshop.

### *In situ* Hybridization

*C. annuum* ‘Bukang’ cotyledons were inoculated with CMV-Fny, P1 and chimeric viruses. At four and eight days after inoculation, inoculated cotyledons were sampled for *in situ* hybridization. Leaf tissues were fixed by vacuum infiltration in 10% formaldehyde, 50% ethanol, and 5% acetic acid buffer and then dehydrated via an ethanol series. Fixed tissues were infiltrated and embedded in Paraplast Plus (Sigma, St Louis, MO, USA). Tissues in paraffin were sectioned into 8 µm thicknesses using an HM 340E rotary microtome (Microm International, Walldorf, Germany). Sections were deparaffinized by xylene (Sigma, St Louis, MO, USA) and incubated with 10 µg/ml proteinase K (Sigma, St Louis, MO, USA) for 15 min at 37°C. To synthesize probe, CMV-Fny and P1 RNA3 were labeled with DIG-nick translation mix (Roche, Basel, Switzerland) according to the manufacturer’s instructions. Sections were then hybridized with DIG-labeled probes for 16 hrs at 42°C. Sections were washed with various dilutions of SSC buffers and incubated with anti-DIG-alkaline phosphatase (Roche, Basel, Switzerland) for 2 hrs at room temperature. The samples were visualized in staining reactions with 4-nitro blue tetrazolium chloride (NBT)/5-bromo-4-chloro-3-indolyl-phosphate (BCIP) solutions (Roche, Basel, Switzerland). *In situ* hybridized samples were detected and photographed by Axiophot light microscopy (Carl Zeiss, Jena, Germany).

### Quantitative Real-time RT-PCR and Calculation of Genomic RNA Copy Numbers

Leaf discs from leaves of *C. annuum* ‘Bukang’ mock-inoculated or inoculated with CMV-Fny, P1, Fny-GFP, P1-GFP, or chimeric viruses were ground in liquid nitrogen. RNA was extracted by TRIzol reagent (Invitrogen, Carlsbad, CA, USA) in accordance with the manufacturer’s protocol. cDNA was synthesized from total RNA template (3 µg) using reverse transcriptase (Promega, Madison, WI, USA). Then, 5 µl of 1∶100 diluted cDNA was used for the real-time RT-PCR reaction. Primer sets (P3-qRT(F/R), F3-qRT2(F/R)) for real-time PCR were designed using the RNA3 sequence (Table S1). To generate a standard curve, PCR products from the primer sets were purified and serially diluted in water (10 ng to 10^−5 ^ng). Real-time PCR was carried out in 20 µl reaction volumes containing 10 mM Tris-HCl (pH8.3), 50 mM KCl, 1.5 mM MgCl_2_, 0.25 mM of each dNTP, 5 pmol of each primer, one unit rTaq polymerase (Takara, Shiga, Japan), 1.25 µM Syto9 (Invitrogen, Carlsbad, CA, USA) and cDNA or template for the standard using a Roter-Gene^TM^6000 thermocyler (Corbett, Mortlake, NSW, Australia). Cycling conditions were 95°C for 4 min, followed by 95°C for 20 sec, 60°C for 20 sec, 72°C for 20 sec for 55 cycles. The target gene amounts from each sample were calculated based on the C_t_ value and ubiquitin gene [37] results in the corresponding samples. Genomic RNA copy numbers were calculated using the following equation: N (copy number of per µl) = C (concentration of samples)/(K (length of target gene)×330×1.6601×10^−18^) [38].

## Results

### CMV-P1 RNA1 Determines Systemic Infection in *C. annuum* ‘Bukang’

**Figure 1:**
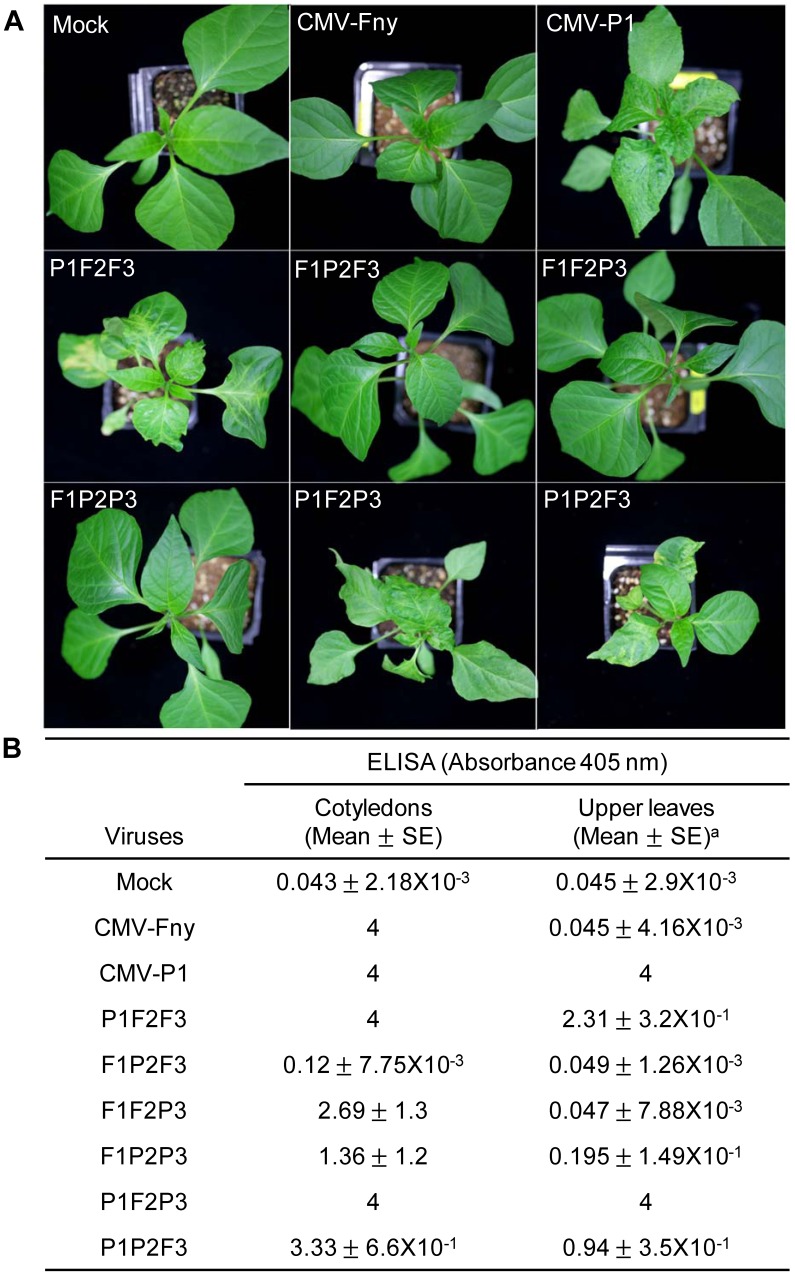
Disease response in *C. annuum* ‘Bukang’ inoculated with CMV-Fny, CMV-P1, and CMV reassortants. **A**, Disease symptoms of systemic leaves inoculated with the indicated viruses (CMV-Fny, CMV-P1, P1F2F3, F1P2F3, F1F2P3, F1P2P3, P1F2P3, and P1P2F3) or mock inoculated. Pepper cotyledons were inoculated using sap from infected *N. benthamiana*. Photographs were taken at 21 days post-inoculation. **B**, Detection of CMV accumulation via enzyme-linked immunosorbent assay (ELISA). Two leaf discs from inoculated cotyledons and the upper leaves were sampled at 28 days post-inoculation. Three independent experiments were each performed in triplicate. ^a^ Average of absorbance value and standard errors.

*C. annuum* ‘Bukang’ is resistant to two CMV strains, CMV-Kor and CMV-Fny, but susceptible to CMV-P1, which infects the plant systemically [32]. To identify which RNA genome segment is responsible for overcoming the *Cmr1*-mediated resistance, reassorted CMV viruses were constructed by combining the CMV-Fny and CMV-P1 cDNA clones. F1, F2, and F3 represent CMV-Fny RNA1, RNA2, and RNA3, while P1, P2, and P3 represent CMV-P1 RNA1, RNA2, and RNA3, respectively. Reassorted viruses F1P2P3, P1F2P3, P1P2F3, P1F2F3, F1P2F3, and F1F2P3, generated by mixing *Agrobacterium* harboring plasmids expressing CMV-Fny and CMV-P1 RNAs**,** were inoculated first to *N. benthamiana* leaves and homogenates of the *N. benthamiana* leaves were inoculated onto ‘Bukang’ and symptom development was monitored. Ten to 12 dpi, mosaic and leaf distortion symptoms started to develop on upper leaves in ‘Bukang’ inoculated with CMV-P1, whereas plants inoculated with CMV-Fny did not show systemic infection (Figure 1A). These findings confirmed our previous results [32].

Three reassortant viruses (P1F2F3, P1F2P3, and P1P2F3) containing the CMV-P1 RNA1 genome caused systemic symptoms, whereas three other reassortants (F1P2F3, F1F2P3, and F1P2P3) did not induce systemic symptoms (Figure 1A). Symptoms of reassortant viruses containing CMV-P1 RNA1 and the original CMV-P1 were slightly different. CMV-P1 induced mosaic symptoms and leaf distortion in systemic leaves. However, the three reassortant viruses (P1F2F3, P1F2P3, and P1P2F3) containing CMV-P1 RNA1 induced more severe leaf distortion and mottling symptoms in systemic leaves than those induced by CMV-P1 (Figure 1A). To test for virus accumulation in cotyledons and upper leaves, we performed ELISA (Figure 1B). The CMV CP was detected in the cotyledons inoculated with F1P2P3, F1F2P3, or F1P2F3, whereas CP was not detected in the upper leaves of the plants inoculated with these reassortants. Accumulation of CP in leaves inoculated with F1F2P3 and F1P2P3 was lower, and that in F1P2F3-inoculated cotyledons was notably reduced compared to that in the CMV-Fny-inoculated equivalents. The CMV CP was detected in both inoculated leaves and upper leaves of the P1F2F3, P1F2P3, and P1P2F3 virus-inoculated ‘Bukang’ plants. Accumulation levels of CP in cotyledons and upper leaves of plants inoculated with P1F2P3 were similar to those of CMV-P1. The accumulation of CP corresponding to two other reassortants (P1F2F3 and P1P2F3) was slightly different. In the case of P1F2F3, the accumulation level of CP in inoculated leaves was similar to that of CMV-P1, whereas the accumulation level of CP in upper leaves was lower than that of CMV-P1. Less accumulation of CP was detected in upper leaves of plants inoculated with P1P2F3 than in those of plants inoculated with CMV-P1 (Figure 1B). Although each CMV reassortant containing CMV-P1 RNA1 showed a different level of CP accumulation, these reassortants showed accumulation of CMV CP in both cotyledons and upper leaves. Taken together, these findings indicate that CMV-P1 RNA1 is a determinant of systemic infection of *C. annuum* ‘Bukang.’

### The C-terminal Region of the Helicase Domain is Responsible for Systemic Infection in *C. annuum* ‘Bukang’

To delimit the sequences in RNA1 controlling systemic infection, the sequences of CMV-Fny RNA1 and CMV-P1 RNA1 were first compared. Sequence analyses showed 90% nucleotide identity and 96% amino acid identity. The 1a proteins of CMV-Fny and CMV-P1 strains differ in 37 of 993 amino acids, with the differences scattered in both domains and the hinge region (Table 1).

**Table 1:** Amino acid differences in protein 1a of CMV-Fny and CMV-P1.

Region	Number of aa differences	Position	CMV-Fny	CMV-P1
1–456	18	23	Thr	Asn
(Methyltransferase		54	Gly	Ser
domain)		147	Asn	Ser
		169	Gln	His
		224	Val	Leu
		242	Thr	Ala
		249	Val	Ser
		252	Leu	Ile
		255	Thr	Ser
		256	Val	Gly
		258	Ser	Thr
		259	Arg	Gly
		266	Met	Leu
		267	Val	Ile
		284	Glu	Lys
		298	Arg	Lys
		310	His	Asn
		355	Glu	Lys
457–645	9	513	Tyr	Phe
		542	Ser	Ile
		550	Thr	Ala
		553	Gln	Pro
		555	Pro	Leu
		566	Arg	Gln
		576	Ala	Val
		585	Val	Ile
		623	Arg	Lys
646–993	10	646	Ile	Val
(Helicase domain)		662	Val	Ala
		690	Cys	Ser
		697	Thr	Ala
		865	His	Arg
		896	Ser	Glu
		901	Ile	Val
		957	Gln	Lys
		980	Val	Ala
		993	Ala	Val

**Figure 2:**
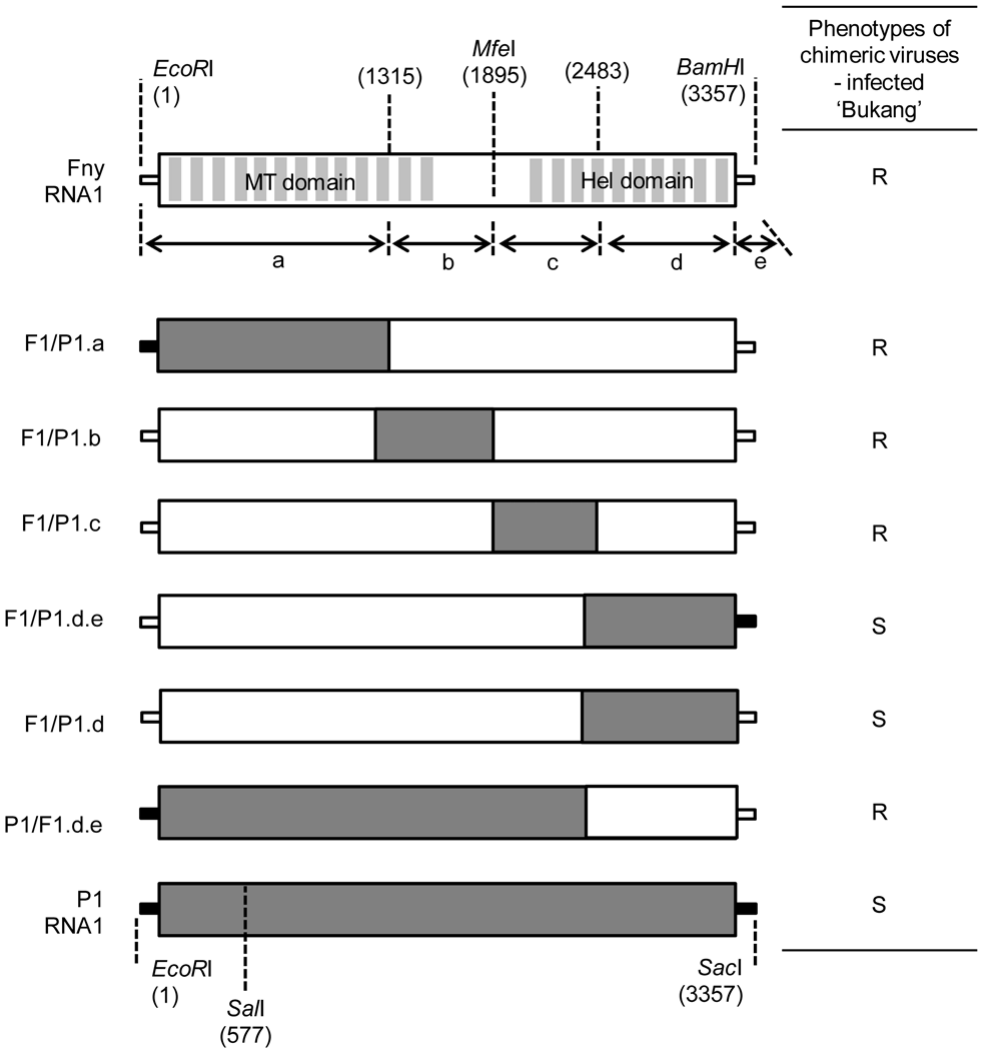
Schematic diagram of CMV RNA1 chimeric viruses. White boxes and gray boxes indicate Fny-derived regions and P1-derived regions, respectively. Dotted lines show the exchange position or the common restriction site between CMV-P1 and CMV-Fny used in the construction of the chimeras. R, resistant; S, susceptible.

**Figure 3:**
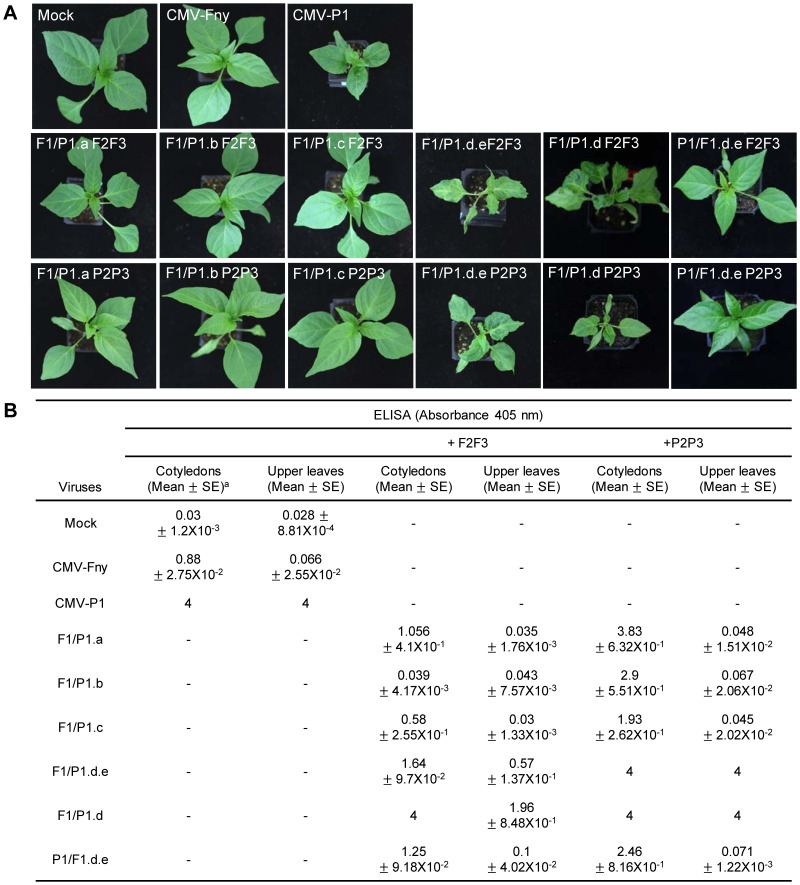
Symptoms in *C. annuum* ‘Bukang’ inoculated with CMV-Fny, CMV-P1, and CMV RNA1 chimeras. A , Symptoms in *C. annuum* ‘Bukang’ inoculated with CMV-Fny, CMV-P1, and CMV RNA1 chimeras. Cotyledons were inoculated using sap from infected *N. benthamiana*. The photographs were taken at 21 days post-inoculation. **B**, Accumulation of CMV coat protein in inoculated leaves and systemic leaves of peppers was detected using ELISA. Two leaf discs of the inoculated cotyledons and the non-inoculated leaves from each plant were sampled at 21 days post-inoculation. Three independent experiments were each performed in triplicate. ^a^Average of absorbance value and standard errors.

Next, four chimeric constructs between CMV-Fny RNA1 and CMV-P1 RNA1 were constructed. CMV-Fny and CMV-P1 RNA1 were divided into four domains, and four chimeric constructs were generated by substitution of genomic regions of CMV-P1 with those of CMV-Fny. The nucleotide sequence regions 1 to 1315, 1293 to 1895, 1896 to 2483, 2468 to 3077 and 3073 to 3357 represent the a, b, c, d and e regions, respectively. The regions spanning nucleotides 1 to 1315 and 1293 to 1895 of CMV-Fny were replaced by the corresponding regions of CMV-P1, resulting in F1/P1.a and F1/P1.b, respectively. F1/P1.c and F1/P1.d.e were constructed in a similar manner (Figure 2). F1/P1.a and F1/P1.b are chimeras in which the methyltransferase domain of CMV RNA1 has been exchanged, while F1/P1.c and F1/P1.d.e are chimeras in which the helicase domain of CMV RNA1 has been exchanged. These chimeric viruses generated from RNA1 molecules were mixed with the RNA2 and RNA3 of either CMV-Fny or CMV-P1 and inoculated onto *N. benthamiana*. At 10–14 dpi, all *N. benthamiana* plants inoculated with the CMV chimeras showed typical CMV symptoms. Extracts were prepared from infected *N. benthamiana* and inoculated onto ‘Bukang.’ Inoculated pepper plants were monitored for three to four weeks. CMV-P1 caused severe symptoms in whole plants, starting at 10–12 dpi in the first true leaves and continuing to develop into upper leaves. At the same time, the chimera containing the 3′ end of P1 RNA, F1/P1.d.e, induced vein-clearing mosaic and leaf-distortion symptoms, whereas CMV-Fny caused no detectable symptoms in the upper leaves of ‘Bukang’ (Figure 3A). To assess CMV accumulation, the inoculated and upper leaves of inoculated plants were sampled at 21 dpi. As can be seen in Figure 3B, accumulation of CMV CP was detected in the inoculated leaves of plants inoculated with CMV-Fny, CMV-P1, and all chimeras except for F1/P1.b.F2F3. The combination of chimeric viruses having CMV-Fny RNA2 and RNA3 (F1/P1.a.F2F3, F1/P1.c.F2F3, and F1/P1.d.e.F2F3) accumulated to lower levels in inoculated leaves compared to chimeric viruses with CMV-P1 RNA2 and RNA3 (F1/P1.a.P2P3, F1/P1.c.P2P3, and F1/P1.d.e.P2P3). The CP of F1/P1.b.F2F3 was not detected in inoculated leaves, but accumulation of CP of F1/P1.b.P2P3 was detected. In upper leaves, the CP was detected in the plants inoculated with CMV-P1, F1/P1.d.e.F2F3, or F1/P1.d.e.P2P3 chimeras. However, F1/P1.d.e.F2F3 had a lower level of CP accumulation than did CMV-P1 or F1/P1.d.e.P2P3 (Figure 3B). The virus F1/P1.d.e also contained the 3′ untranslated region (UTR) of CMV-P1 RNA1. In order to determine whether the 3′ UTR influences systemic infection, we constructed F1/P1.d by combining the CMV-P1(2468-3077) coding sequence with the CMV-Fny 3′ UTR (3077–3357) (Figure 2). At 21 dpi, ‘Bukang’ infected by F1/P1.d showed symptoms similar to those of F1/P1.d.e -infected plants (Figure 3A). We generated a reciprocal P1/F1.d.e chimera by inserting the CMV-Fny RNA1 sequence from 2468 to 3357 into the CMV-P1 RNA1 sequence (Figure 2). The chimera P1/F1.d.e did not cause systemic infection by 21 dpi (Figure 3A). These results suggest that differences between CMV-Fny and CMV-P1 in the sequence from 2468 to 3077 conferred the ability of CMV-P1 to cause systemic infection in *C. annuum* ‘Bukang.’ The region containing nucleotides 2468 to 3077 in CMV RNA1 encodes amino acids 792 to 993 of CMV protein 1a, located at the C-terminus of the helicase domain. Taken together, these results suggest the C-terminus of the helicase domain is a determinant for systemic infection of *C. annuum* ‘Bukang.’

### Four Amino Acid Substitutions in the Helicase Domain Convert Avirulence to Virulence in the *Cmr1* Genotype Pepper

**Figure 4:**
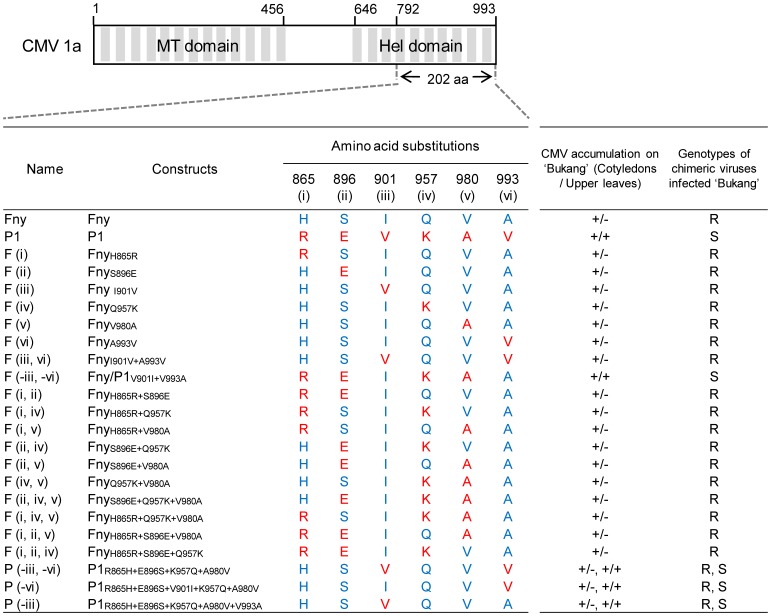
Schematic diagram of CMV-Fny- and CMV-P1-derived amino acid substitution mutants. The positions of the amino acid substitutions are located at the top of each amino acid sequence. Fny- and P1-derived amino acids are indicated in blue and red, respectively. At 21 days post-inoculation, accumulation of CMV was detected by ELISA. +, presence of virus; −, absence of virus; R, resistant; S, susceptible.

Sequence analysis showed that the 202 amino acid region between amino acids 792 and 993 has six amino acid differences between CMV-Fny and CMV-P1 (Figure 4). To identify the amino acids involved in systemic infection, the nucleotide sequences encoding each of the six amino acids from CMV-Fny were replaced individually with the nucleotide sequences encoding the corresponding amino acid from CMV-P1 (Figure 4). Cotyledons of ‘Bukang’ were infected with each of the six point-mutant viruses, F (i), F (ii), F (iii), F (iv), F (v), and F (vi). Phenotypic changes in the inoculated plants were observed for 21 to 28 dpi. All mutant viruses replicated competently and caused typical CMV systemic symptoms in *N. benthamiana*. However, none of these mutants induced symptoms in the upper leaves of ‘Bukang’ (Figure 4). Although a small number of ‘Bukang’ plants infected with F (iii) or F (vi) showed slower growth than plants inoculated with other substitution mutants, accumulation of CP was not detected in the upper leaves. A few studies have reported that alterations of virulence factors generally involve more than two amino acid changes [39], [40]. Thus, we hypothesized that more than two amino acids in the C-terminal region of the helicase domain (202 amino acids) were required for systemic infection in ‘Bukang’ plants. First, we divided the six amino acids into two groups based on the phenotypic results. In the results from the single amino acid substitution mutants, plants infected with F (iii) or F (vi) showed slower growth than those infected with other mutants. Therefore, the two amino acids, Val at the 901 and 993 positions, were classified as group I, and the four other amino acids were classified as group II. We constructed a group I mutant virus in which the nucleotide sequences encoding the CMV-Fny 1a protein amino acids were replaced with the corresponding sequences encoding CMV-P1 1a protein amino acid residues at positions 901 and 993. A group II mutant virus was constructed in which the CMV-P1 1a protein amino acids at positions 901 and 993 were replaced with those of CMV-Fny based on the F1/P1.d.e construct. Group I and group II mutants were designated F (iii, vi) and F (-iii, -vi), respectively (Figure 4), and inoculated onto ‘Bukang’ cotyledons. At 21 dpi, CMV-P1 and F (-iii, -vi) induced systemic symptoms whereas CMV-Fny and F (iii, vi) did not. F (-iii, -vi) induced milder symptoms than did CMV-P1 (Table 2). Accumulation of CP was detected in the inoculated leaves of all mutant-infected ‘Bukang.’ In upper leaves, CP was detected in plants infected with CMV-P1, F (-iii, -vi) F2F3, or F (-iii, -vi) P2P3. F (-iii, -vi) with CMV-Fny RNA2 and RNA3 showed a lower level of CP accumulation than did F (-iii, -vi) with CMV-P1 RNA2 and RNA3 in inoculated and upper leaves (Table 2). These results suggest that more than one of the four amino acid changes present in CMV-P1 (865, 896, 957, and 980) is necessary for systemic infection in ‘Bukang.’

**Table 2:** Summary of experiments with helicase domain mutant viruses.

	No. of systemic infected plants^a^				ELISA (Absorbance 405nm)^c^	
Viruses	10 dpi	16 dpi	21 dpi	Infection phenotype on upper leaves^b^	Cotyledons (Mean ± SE)^d^	Upper leaves (Mean ± SE)
CMV-Fny	0/15	0/15	0/15	N	0.80±6.67×10^−4^ e	0.05±5.57×10^−4^
CMV-P1	14/14	14/14	14/14	LD, M, SG	3.92±5.56×10^−3^	3.72±3.35×10^−2^
F (iii, iv)	0/15	0/15	0/15	N	4.00	0.04±9.19×10^−4^
F (-iii, -iv)	15/15	15/15	15/15	MLD, M, SG	3.86±1.14×10^−1^	2.85±4.02×10^−2^
P (-iii, -vi)	0/20	7/20	10/20	MLD, MM	1.66±9.54×10^−2^	1.22±7.28×10^−2^
P (-vi)	0/10	1/10	1/10	MLD, MM	–	–
P (-iii)	0/10	2/10	2/10	MLD, MM	–	–

a Systemically infected plants/total number of plants tested.

b Abbreviations of infection phenotypes = N, none; LD, leaf distortion; M, mosaic; SG, stunted growth; MLD, mild leaf distortion; MM, mild mosaic.

c The inoculated cotyledons and the upper leaves of inoculated plants were sampled at 21 dpi. Mean values from triplicates samples are given.

d Average of absorbance value and standard errors.

e Mean of values in triplicate.

In order to identify which among the four amino acids (positions 865, 896, 957, and 980) are required for systemic infection, ten amino acid substitution mutants were designed, each with two or three changes from the CMV-P1 amino acid sequence introduced into the CMV-Fny sequence (Figure 4). Six mutants contain two replacement amino acids and four mutants contained three replacement amino acids: F (i, ii), F (i, iv), F (i, v), F (ii, iv), F (ii, v), F (iv, v), F (ii, iv, v), F (i, iv, v), F (i, ii, v), and F (i, ii, iv), respectively. These ten mutants were inoculated onto ‘Bukang’ cotyledons along with CMV-P1 RNA2 and RNA3. Phenotypic changes were monitored for four weeks post-inoculation. However, none of the mutants induced symptoms in the non-inoculated upper leaves. CP of the mutant viruses was detected only in the inoculated leaves of ‘Bukang’ (Figure 4). These results show that the amino acids derived from the CMV-P1 sequence that are crucial for systemic infection may be the four amino acids at positions 865, 896, 957, and 980.

### Four Amino Acid Substitutions Affect for Cell-to-cell Movement in Inoculated Leaves

**Figure 5:**
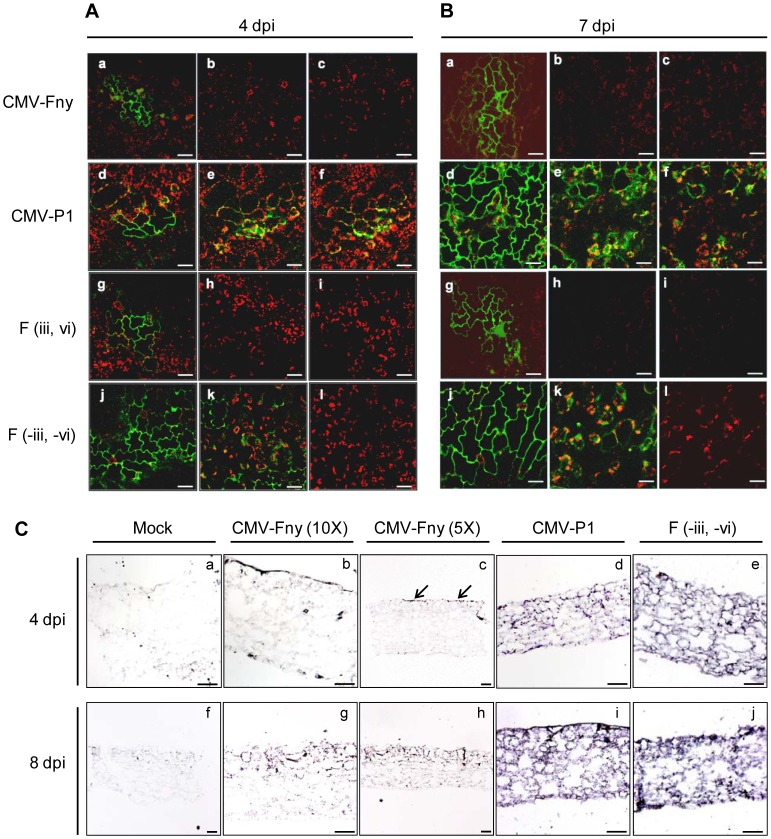
Virus movement and localization studies with CMV clones. **A and B**, Localization of CMV-Fny-GFP (A–a, b, c; B–a, b, c), CMV-P1-GFP (A–d, e, f; B–d, e, f), F (iii, vi)-GFP (A–g, h, i; B–g, h, i), and F (-iii, -vi)-GFP (A–j, k, l; B–j, k, l) in *C. annuum* ‘Bukang.’ Images a to c, d to f, g to i, and j to l in panels A and B indicate optical sections moving down into the leaf from the epidermal cells at the leaf surface to the underlying mesophyll cells. GFP fluorescence was visualized by confocal laser scanning microscopy at 4 (A) and 7 (B) days post-inoculation (dpi). Green indicates GFP fluorescence and red indicates chlorophyll autofluorescence. Scale bars = 50 µm. **C**, *In situ* hybridization localization of the CMV-Fny, P1 and F (-iii, -iv) in inoculated leaves of *C. annuum* ‘Bukang’ at 4 dpi (C–a to e) and 8 dpi (C–f to j). These images are transverse sections of mock (a and f)-, CMV-Fny (b, c, g and h)-, CMV-P1 (d and i)- and F (-iii, -iv) (e and j)-inoculated leaf tissues. The sections of CMV-Fny inoculated leaves shows two scale images, 10X (b and g) and 5X (c and h). Purple color and arrows indicate the infected region. Scale bars = 100 µm.

To observe systemic infection of various CMV constructs at a cellular level, CMV-Fny-GFP, CMV-P1-GFP, F (-iii, -vi)-GFP, and F (iii, vi)-GFP were inoculated onto ‘Bukang’ cotyledons, and the GFP signal was observed in the inoculated leaves by confocal laser scanning microscopy at 4 and 7 dpi. Whereas the fluorescence of CMV Fny-GFP and F (iii, vi)-GFP was detected only in epidermal cells (Figure 5A a), the GFP signal of CMV-P1-GFP and F (-iii, -vi)-GFP was observed in mesophyll cells as well as epidermal cells (Figure 5A d, e, f, j and k). At this time point, the F (-iii, -vi)-GFP signal in the epidermal cells was distributed to more cells than that of the three other virus constructs [CMV-Fny-GFP, CMV-P1-GFP, and F (iii, vi)-GFP]. At 7 dpi, horizontal movement of the GFP signal from the site of infection to adjacent epidermal cells was observed in all GFP-viruses (Figure 5B). However, CMV-Fny-GFP (Figure 5B a, b, and c) and F (iii, vi)-GFP (Figure 5B g, h, and i) were observed only in epidermal cells. By contrast, CMV-P1-GFP and F (-iii, -vi)-GFP moved from the epidermal cells into mesophyll cells (Figure 5B d, e, f, j, k, and l). When we visualized the movement of CMV-P1-GFP and F (-iii, -vi)-GFP in inoculated leaves of *Cmr1* plants at 7 dpi, CMV-P1-GFP spread deeper into the mesophyll cell layers than did F (-iii, -vi)-GFP. The time course of movement was slightly different between CMV-P1-GFP and F (-iii, -vi)-GFP. However, there seemed to be no direct effect on the severity of systemic infection.

To investigate further the cell-to-cell movement of various CMV constructs, we performed *in situ* hybridization with CMV RNA3 probes on cross sections of inoculated leaves. Cotyledons of ‘Bukang’ inoculated with CMV-Fny, CMV-P1 or F (-iii, -vi) were sampled at 4 dpi and 8 dpi. CMV-Fny and P1, F (-iii, -vi)-inoculated samples were hybridized with CMV-Fny RNA3 and CMV-P1 RNA3 as probes, respectively. Mock-inoculated samples were also hybridized with both RNA3s. Hybridization signal was not detected on sections of mock-inoculated samples at 4 and 8 dpi (Figure 5C a and f). Weak and spotty hybridization signals were observed in epidermal cells of CMV-Fny-inoculated leaves at 4 dpi (Figure 5C b and c). Even though weak signals were observed under epidermal cells, hybridization signals were mostly observed in epidermal cells of CMV-Fny-inoculated leaves at 8 dpi (Figure 5C g and h). Hybridization signals were observed in both epidermal and mesophyll cells of CMV-P1 and F (-iii, -vi)-inoculated leaves at 4 and 8 dpi (Figure 5C d, e, i and j). These GFP and *in situ* hybridization results demonstrate that four amino acid substitutions are responsible for cell-to-cell movement of CMV in *Cmr1* plants.

### Differential Replication of CMV-Fny and CMV-P1 in Inoculated Leaves

Quantitative real time RT-PCR was carried out to check the levels of virus replication in inoculated leaves. *C. annuum* ‘Bukang’ cotyledons were inoculated with CMV-Fny, CMV-P1 or chimeric viruses [F (iii, vi) and F (-iii, -vi)]. Virus replication was quantified by real time RT-PCR using an equation reported previously [38]. Cotyledons inoculated with each virus were sampled from three independent plants at 4, 7 and 10 dpi. CMV RNA was detected in all samples except for the mock-inoculated samples. Quantification data showed that the copy numbers of CMV RNA at 10 dpi were significantly increased in inoculated plants for all tested virus constructs compared to those at 4 dpi. The CMV-Fny RNA copy number at 4 dpi was 3.55×10^4^ per 150 ng of total RNA, whereas the CMV-P1 copy number was 1.64×10^6^ per 150 ng of total RNA, nearly 50-fold higher. At 10 dpi, RNA accumulation of CMV-Fny and -P1 were increased by 1.2-fold and 8.5-fold, respectively, compared to the corresponding infection at 4 dpi (Table 3). The copy number increment of RNA CMV-Fny from 4 to 10 dpi was very minimal. These results show that replication of CMV-Fny is less efficient than that of CMV-P1 in *C. annuum* ‘Bukang.’ To examine the effect on replication of four amino acid substitutions, CMV accumulation of F (iii, vi) and F (-iii, -vi) were also quantified at 4, 7 and 10 dpi. At 4 dpi, F (-iii, -vi) showed more RNA copies than did F (iii, vi). At 10 dpi, the numbers of RNA copies of F (iii, vi) and F (-iii, -vi) were increased by 4.5-fold and 2.7-fold, respectively, compared to the corresponding infections at 4 dpi. Even though F (iii, vi) infections showed a 4.5-fold increase between 4 and 10 dpi, there were fewer copies of F (iii, vi) than of F (-iii, -vi) and CMV-P1 at the corresponding time points (Table 3). Altogether, these results demonstrate that four amino acids are crucial for replication of CMV-P1 in *C. annuum* ‘Bukang.’

**Table 3:** Quantification of the genomic RNA copies CMV-Fny, CMV-P1 and chimeric viruses in inoculated leaves of *C. annuum* ‘Bukang’.

	No. of genomic RNA copies ± SE^a^		
Viruses	4 dpi	7 dpi	10 dpi
Mock	ND^b^	ND	ND
CMV-Fny	3.55×10^4^±1.35×10^3^	5.23×10^4^±6.43×10^3^	4.34×10^4^±4.76×10^3^
CMV-P1	1.64×10^6^±5.77×10^4^	7.48×10^6^±2.59×10^6^	1.39×10^7^±2.14×10^6^
CMV-Fny-GFP	8.98×10^4^±2.35×10^4^	9.40×10^4^±2.32×10^3^	9.12×10^4^±2.19×10^4^
CMV-P1-GFP	5.77×10^6^±6.69×10^5^	1.22×10^7^±5.93×10^5^	1.15×10^7^±2.22×10^6^
F (iii, iv)	6.69×10^4^±4.93×10^3^	1.39×10^5^±5.69×10^4^	3.03×10^5^±1.81×10^4^
F (-iii, -iv)	2.70×10^6^±3.06×10^5^	3.28×10^6^±9.21×10^5^	7.32×10^6^±8.23×10^5^

a Average number of genomic RNA copies per 150 ng of total RNA and standard errors.

b Not detected.

### Achieving Complete Loss of Virulence in CMV-P1 Requires Six Amino Acid Mutations in the Helicase Domain

The F (iii, vi) mutant could not infect the upper leaves of *C. annuum* ‘Bukang.’ To determine the virulence in *C. annuum* ‘Bukang’ of CMV-P1 mutants, P (-iii, -vi), was constructed (Figure 4). At 10 dpi, CMV-Fny- and P (-iii, -vi)-inoculated plants did not show systemic symptoms on the first true leaves, whereas CMV-P1-inoculated plants did. However, P (-iii, -vi)-inoculated plants showed symptoms in seven out of 20 plants at 16 dpi (Table 2). At 21 dpi, CMV symptoms developed in three other plants. A total of ten out of 20 plants were infected. Even though the plants were systemically infected by P (-iii, -vi), the symptoms of these plants were milder than those of CMV-P1 infected plants. To determine the levels of CMV accumulation in P (-iii, -vi)-inoculated plants, ELISA was performed at 21 dpi. In CMV-Fny-infected plants, coat protein was detected only in inoculated cotyledons. CMV-P1-infected plants consistently showed high levels of coat protein in both inoculated cotyledons and upper leaves (Table 2). Systemically infected plants of P (-iii, -vi) showed lower levels of coat protein accumulation compared to plants infected with CMV-P1 (Table 2), whereas plants that were not infected systemically showed no coat protein accumulation in upper leaves (data not shown). These results demonstrate that P (-iii, -vi) mutants have less virulence compared to CMV-P1. To test the effect of more amino acid mutations on virulence, P (-vi) and P (-iii) were constructed. P (-vi) and P (-iii) contain five CMV-Fny-derived amino acid mutations in the CMV-P1 background (Figure 4). Among P (-vi)- and P (-iii)-inoculated plants, only one or two plants were infected at 16 and 21 dpi, respectively (Table 2), and these systemically-infected plants also showed milder symptoms. These results show that variants with five amino acid mutations still retain minimal virulence. However, P1/F1.d.e, which contains six CMV-Fny-derived amino acid mutations in the CMV-P1 background, completely lost virulence in ‘Bukang’ (Figure 2).

## Discussion

In this study, we demonstrated that changes in the helicase domain of RNA1 permit CMV-P1 to overcome the *Cmr1* resistance in peppers. A systematic approach using various chimeric virus constructs and mutant viruses further revealed that four amino acid substitutions in the helicase domain at positions 865, 896, 957, and 980 play a central role in determining systemic infection in *C. annuum* ‘Bukang,’ which has the *Cmr1* gene and is thus resistant to CMV-Fny.

Prior studies have shown that avirulence/virulence factors, symptom determinants, and systemic infection of CMV are influenced by RNA1. Two studies reported that CMV RNA1, CMV 1a protein residue 461, was directly implicated as a virulence determinant [13], [41]. Although RNA1 was not directly implicated as an avirulence determinant, other reports have shown that RNA1 is involved in determining the severity of symptoms [42], [43]. These studies reported that key domains of the symptom severity factors in CMV RNA1 are distributed evenly throughout the RNA1 sequence, except for in the helicase domain. Yamaguchi et al. (2005) reported that the avirulence factor of a CMV-HL isolate contained a partial helicase sequence, but the 5′ UTR and the hinge region between the methyltransferase and helicase domains also controlled systemic infection in lily [44]. Our study demonstrates that only alterations in the helicase domain of CMV were necessary for systemic infection.

The CMV helicase domain is involved in replication of the virus. Deletion of the helicase domain or fusion of a protein to the helicase domain prevents virus replication [4]. In this study, we constructed chimeric and mutant viruses using the helicase domain sequence. Although mutations in the helicase domain could have resulted in an inability of the virus to infect plants, these mutant viruses accumulated normally in both inoculated and upper leaves and induced mosaic symptoms on upper leaves of *N. benthamiana* and *C. annuum* ‘Jeju.’ These results indicate that the replacement of amino acids does not significantly affect the replication function of the helicase domain.

Our observation that F (-iii, -vi) infected systemic leaves of *C. annuum* ‘Bukang’ suggests that amino acids 865, 896, 957 and 980 are involved in systemic infection. However, the P (-iii, -vi), P (-vi) and P (-iii) mutants containing four or five amino acids (865, 896, 901, 957, 980 or 865, 896, 957, 980, 993) derived from CMV-Fny in the CMV-P1 background could infect systemic leaves (Table 2). This result indicates that the reverse mutant [P (-iii, -vi)] does not confer complete avirulence. Instead, P (-iii, -vi) appears to be associated with delayed development of symptoms and mild symptoms. Two other reverse mutants containing five CMV-Fny-derived amino acids, P (-vi) and P (-iii), also showed very weak symptoms in a small number of plants. Only the P1/F1.d.e construct restored avirulence in *Cmr1* pepper. This result suggests that the four amino acids from CMV-P1 are related to virulence and the six amino acids from CMV-Fny are related to avirulence. Such a discrepancy between avirulence and virulence factors is rather unusual [44]. However, virulence and avirulence factors of *Soybean mosaic virus* (SMV) are also not the same [40]. One amino acid of SMV was associated with loss of virulence, and three amino acids of SMV were related to gain of virulence [40].

Inhibition of long-distance movement is a common CMV resistance mechanism [31], [32], [45], [46], [47], [48]. Some avirulence factors of CMV play a role in the inhibition of local and systemic movement in host plants [15], [24], [25], [49], [50], [51]. However, these reports explained the mechanism using phenotypic data. Cellular observations of virus movement in relation to avirulence factors are rare in CMV. Thus, we observed the effects of the avirulence factors at the cellular level. The fluorescence of CMV-P1-GFP and F (-iii, -iv)-GFP was detected in epidermal and mesophyll cells, whereas CMV-Fny-GFP and F (iii, iv)-GFP were detected only in the epidermal cell layers. These results indicated that CMV-Fny-GFP and F (iii, iv)-GFP failed to enter the mesophyll cells of inoculated leaves and that these two viruses were not able to enter the vascular tissue to initiate systemic movement. On the other hand, CMV-P1-GFP and F (-iii, -iv)-GFP seemed to move from the systemic leaves through infected epidermal and mesophyll cells to the vascular tissue in inoculated leaves. Furthermore, *in situ* hybridization analysis of transverse views revealed that CMV-P1 and its mutants were present in epidermal and mesophyll cells, whereas CMV-Fny was observed only in the epidermal cell layer. This result of cell-to-cell movement is consistent with our previous work, in which CMV-Fny-GFP was detected only in epidermal cells of inoculated ‘Bukang’ leaves by three dimensional confocal laser microscopy [32]. *In situ* hybridization methods have been used for detection of virus movement including of CMV [52], [53], [54], [55], [56]. The examination of transverse views using *in situ* hybridization is rare in pepper research, however.

In previous studies, it was suggested that a difference in the rate of virus accumulation was associated with virulence of CMV [15], [20]. One of these studies reported that the differential rate of systemic symptom development in squash was due to a difference in CMV replication [15]. In addition, the key factor was RNA1, which also was correlated with different rates of systemic movement [15]. Another study suggested that accumulation of RNA for 2b protein was associated with virulence and systemic symptoms in tobacco [20]. In our study, to compare CMV-Fny and CMV-P1 viral replication and RNA accumulation, RNA copy numbers were quantified by quantitative real-time RT PCR (Table 3). The rate of increase in RNA accumulation of CMV-P1 was higher than those of CMV-Fny and F (iii, vi) in inoculated leaves. Even though the increase rate of F (-iii, -vi) RNA copies showed lower level than that of F (iii, vi) between 4 and 10 dpi, there were more copies of F (-iii, -vi) than that of F (iii, vi) at the corresponding time points. Therefore, our results suggest that four amino acid substitutions from CMV-P1 are not only necessary for cell-to-cell movement in inoculated leaves of the *Cmr1* pepper plants but also influence replication of the virus.

The response of a host plant to viral infection is induced by interactions between host factors and viral factors. Several host factors interacting with CMV 1a have been reported based on the yeast two-hybrid system. One of the identified host factors in tobacco, a plant cellular factor (NtTLP1), regulates CMV replication and movement [57]. Other host factors (Tcoi1 and Tcoi2) in tobacco interacts with the MT domain of CMV 1a [58]–[59]. Tonoplast proteins in *Arabidopsis* (TIP1 and TIP2) affect CMV replication [60]. However, host factors interacting with the helicase domain have not been identified. Host factors may be involved in the systemic infection of CMV in peppers. Therefore, to understand the mechanism of CMV infection in peppers, identification of host factors interacting with the helicase domain will be required.

In conclusion, our study demonstrated that the helicase domain of CMV-P1 affects systemic infection, and four amino acids at the C-terminal end of the helicase domain are responsible for this phenotype in *Cmr1* genotype peppers. *Cmr1* inhibits systemic infection through prevention of cell-to-cell movement, but CMV-P1 containing changes at positions 865, 896, 957, and 980 was able to infect systemic leaves through movement from epidermal cells to mesophyll cells. However, the protein structure of the helicase domain did not differ significantly between CMV-Fny and CMV-P1. Since the interactions between viral proteins and host proteins are necessary for viral infection, isolation of host factors related to avirulence factors would lead to an understanding of the viral resistance gene and avirulence mechanisms in peppers.

## Supporting Information

Table S1 (DOC)
