# Supplementary material for: Helicase Domain Encoded by Cucumber mosaic virus RNA1 Determines Systemic Infection of Cmr1 in Pepper
Source: PLoS One. 2012 Aug 15;7(8):e43136. doi: 10.1371/journal.pone.0043136 (PMC3419664; doi:10.1371/journal.pone.0043136)
Supplement: Table S1 — (DOC) [file pone.0043136.s001.doc]

Table S1. Oligonucleotides used in this study

| Oligomer name | Sequence (5’-3’) | Purpose |
| --- | --- | --- |
| CMV-R1R2-5′-BamHI-Fw | AGGGATCCGTTTATTTACAAGAGCGTACGG | To construct infectious cDNA clones of CMV-P1 |
| CMV-3′-BamHI-Rv | AGGGATCCTGGTCTCCTTTTRGAGRCC |  |
| CMV-R3-5′-BamHI-Fw | AGGGATCCGTAATCTTACCACTGTG |  |
| F1/P1-5’UTR-EcoRI | CGGAATTCGTTTATTTACAAGAGCGTACGG | To construct chimeric viruses |
| FNY-3’UTR-BamHI | CGGGATCCTGGTCTCCTTTTAGAGACCCC |  |
| F1-MfeI (F) | CGCAATTGCACGACACTACAGTGTCTAATTTAA |  |
| P1-MfeI (F) | CGCAATTGCATGACACCACGGTGTCCAACC |  |
| F1/P1-1314DS (F) | ACCATTGTTATTAATGGTATGTC |  |
| P1/P1-1314DS (R) | GACATACCATTAATAACAATGGT |  |
| F1/P1-2482DS (F) | CAATTATGTGCCGTCATGTCT |  |
| F1/P1-2482DS (R) | CACTTTGGTCAATTATGTGCCGTC |  |
| P1-SalI (F) | ACGCGTCGACAGCCGGATTTTTGCACTCA |  |
| FNY-3’UTR(F) | TTAGCGGTCTCCCTCTTCGGGCGGG |  |
| P1-3’ORF (R) | CGAGTGTGTTGCTCGTGTTTAGCGGT |  |
| FNY-3’SacI (R) | CGCGAGCTCTGGTCTCCTTTTAGAGACCCC |  |
| P1-MT (F) | ATGGCGACGTCCTCGTTCAACAT |  |
| P1-MT (R) | CAACGCGGCGAACCACTTCG |  |
| P1-Hel (F) | GTTCATTTCTCCACAGGTCGGT |  |
| P1-Hel (R) | CTAAACACGAGCAACACACTCG |  |
| fH865R (F) | TCCGAAAGGAACCCGCTCAAAATACACGA^a^ | To construct amino acid substitution mutants |
| fH865R (R) | TCGTGTATTTTGAGCGGGTTCCTTTCGGA |  |
| fS896E (F) | GTTGACTTGGATGAGTCCAGGTTCTAT |  |
| fS896E (R) | ATAGAACCTGGACTCATCCAAGTCAAC |  |
| fI901V (F) | TCCAGGTTCTATGTCACGATGACCCAA |  |
| fI901V (R) | TTGGGTCATCGTGACATAGAACCTGGA |  |
| fQ957K (F) | TGACCTGTTTAAAAAATTTTCTTATTGTC |  |
| fQ957K (R) | GACAATAAGAAAATTTTTTAAACAGGTCA |  |
| fV980A (F) | CGAGTACTGTGGTGCATTGAACGGCGATT |  |
| fV980A (R) | AATCGCCGTTCAATGCACCACAGTACTCG |  |
| fA993V (F) | ATGTGTTGCTCGTGTTTAGCGGTCTCCCT |  |
| fA993V (R) | AGGGAGACCGCTAAACACGAGCAACACAT |  |
| pV901I (F) | TCTAGATTTTACATAACGATGACACAA |  |
| pV901I (R) | TTGTGTCATCGTTATGTAAAATCTAGA |  |
| pV993A (F) | GTGTGTTGCTCGTGCTTAGCGGTGTCCCT |  |
| pV993A (R) | AGGGACACCGCTAAGCACGAGCAACACAC |  |
| pR865H (F) | CTTCCGAAAGGAACCCATTCAAAGTATACGAAA |  |
| pR865H (R) | TTTCGTATACTTTGAATGGGTTCCTTTCGGAAG |  |
| pE896S (F) | GTTGATTTAGATTCTTCTAGATTTTAC |  |
| pE896S (R) | GTAAAATCTAGAAGAATCTAAATCAAC |  |
| pK957Q (F) | GACCTGTTCAAGCAGTTTTCTTACTGT |  |
| pK957Q (R) | ACAGTAAGAAAACTGCTTGAACAGGTC |  |
| pA980V (F) | GAGTATTGTGGTGTATTGAACGGCGAT |  |
| pA980V (R) | ATCGCCGTTCAATACACCACAATACTC |  |
| P3-qRT(F) | CCAAGGTACCAGCAGGACTTTAACT | To perform quantitative real time RT-PCR |
| P3-qRT(R) | CTTTAGAGCACAATTCACCAGCATC |  |
| F3-qRT2(F) | CCAAGGTACCAGTAGGACTTTAA |  |
| F3-qRT2(R ) | CTTTTGAGCATAATTCACCAACATC |  |
| CaUBQ-qF1 | CGTGGTGGTTTTTAAGATGATG |  |
| CaUBQ-qR1 | AAAGAAACACAAGGGAACAGAAA |  |
| F3-orf(F) | ATGGCTTTCCAAGGTACCAGTAGGAC | To construct probe for *in situ* hybridization |
| P3-orf(F) | ATGGCTCTCCAAGGTACCAGCAG |  |
| F3,P3-orf(R ) | CTAAAGACCGTTAACCACCTGCGGT |  |

^a^ The mutated sequences for amino acid substitution mutants are underlined.
